# Nucleic Acid Aptamers: Emerging Applications in Medical Imaging, Nanotechnology, Neurosciences, and Drug Delivery

**DOI:** 10.3390/ijms18112430

**Published:** 2017-11-16

**Authors:** Pascal Röthlisberger, Cécile Gasse, Marcel Hollenstein

**Affiliations:** 1 Institut Pasteur, Department of Structural Biology and Chemistry, Laboratory for Bioorganic Chemistry of Nucleic Acids, CNRS UMR3523, 28, rue du Docteur Roux, 75724 Paris CEDEX 15, France; pascal.rothlisberger@pasteur.fr; 2 Institute of Systems & Synthetic Biology, Xenome Team, 5 rue Henri Desbruères Genopole Campus 1, University of Evry, F-91030 Evry, France

**Keywords:** aptamers, systematic evolution of ligands by exponential enrichment (SELEX), modified triphosphates, medical imaging, drug delivery, gene regulation, DNA origami, neurodegenerative diseases

## Abstract

Recent progresses in organic chemistry and molecular biology have allowed the emergence of numerous new applications of nucleic acids that markedly deviate from their natural functions. Particularly, DNA and RNA molecules—coined aptamers—can be brought to bind to specific targets with high affinity and selectivity. While aptamers are mainly applied as biosensors, diagnostic agents, tools in proteomics and biotechnology, and as targeted therapeutics, these chemical antibodies slowly begin to be used in other fields. Herein, we review recent progress on the use of aptamers in the construction of smart DNA origami objects and MRI and PET imaging agents. We also describe advances in the use of aptamers in the field of neurosciences (with a particular emphasis on the treatment of neurodegenerative diseases) and as drug delivery systems. Lastly, the use of chemical modifications, modified nucleoside triphosphate particularly, to enhance the binding and stability of aptamers is highlighted.

## 1. Introduction

Over the last decades, the repository of genetic information in living organisms—DNA—and the vector for gene expression—RNA—have seen an impressive expansion in applications that substantially deviate from their natural functions. Indeed, nucleic acids play a key role in the development of gene silencing therapeutic agents [1,2], the construction of novel nanomaterials [3], and the crafting of biocatalysts [4,5,6]. In this context, aptamers are rapidly developing nucleic acid tools that consist of single stranded DNA or RNA molecules comprising 20–100 nucleotides [7], and that are capable of selective binding to a broad array of targets with remarkable affinity [8,9]. Hence, these functional nucleic acids are often considered as the nucleic acid equivalent of protein antibodies. However, unlike their proteinaceous counterparts, aptamers are not plagued by physical or chemical instability or by potential immunogenicity and can be produced on a relatively large scale by standard chemical synthesis with little batch-to-batch variation [10]. Even though natural aptamers interacting with RNA polymerases have recently been identified [11], these functional nucleic acids are generally isolated in vitro by a combinatorial method coined SELEX (Systematic Evolution of Ligands by Exponential Enrichment) [9,12,13]. During SELEX, large libraries of oligonucleotides (typically 10^14^–10^15^ individual molecules) are challenged to bind to the intended target and iterative rounds of selection-amplification cycles are utilized to enrich the populations with high binding species. Since the invention of the SELEX protocol in the early 1990s, thousands of aptamers have been selected for targets ranging from small molecules [14] to larger entities such as proteins [15,16] or cells [17,18,19], and databases have been created to canalize this exponential growth of aptameric sequences and wealth of information [20,21,22]. The binding capacity of an aptamer is best described by its dissociation constant *K*_d_, which in turn is given by the ratio of the dissociation and association rate constants (*k*_off_/*k*_on_) [23]. Typically, values in the low nM or even pM range are observed for potent aptamers. These impressive properties are reflected by the numerous clinical trials involving aptamers and the first FDA-approved oligonucleotide-based drug (Macugen^®^) [24,25]. Moreover, the versatility of the selection protocol and the high binding affinities have propelled aptamers in the forefront of numerous applications including for instance biosensing [26,27], proteomics [28], purification and biotechnology [29,30,31], therapeutics [25,32], and diagnostics [33,34]. Herein, we have chosen to give an overview and a brief description of the emerging but rapidly growing applications of aptamers. Particularly, we will discuss recent implications of aptamers as radiopharmaceutical tools for medical imaging purposes (MRI and PET imaging) and in neurosciences for the treatment and detection of Alzheimer’s and Parkinson’s diseases. We also discuss the combined use of aptamers with DNA origamis to develop novel nanomaterials and biosensing platforms. Since the development of all these new therapeutic, imaging, and sensing agents require means of targeted delivery, we also cover the use of aptamers as drug delivery systems and as gene silencing agents. The last facet of this review will involve a discussion on the possibility of using chemical modifications to enhance the general properties of aptamers and we will focus particularly on the direct use of modified nucleoside triphosphates (dN*TPs) in SELEX experiments.

## 2. Medical Imaging (MRI and PET)

The ease of chemical modification at both 3′- and 5′-ends [35] combined with the high target affinity and selectivity dramatically increases the potential of aptamers to serve as molecular imaging agents, particularly for magnetic resonance imaging (MRI) and positron emission tomography (PET) [36].

### 2.1. Aptamers and MRI

**Figure 1:**
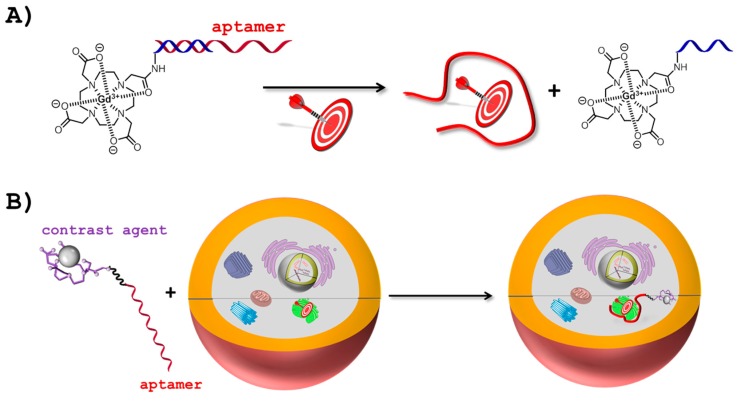
Strategies for the construction of aptamers acting as smart contrast agents: (**A**) Response to a biochemical stimuli: An oligonucleotide is equipped with a Gd^3+^-DOTA complex. This oligonucleotide is complementary to part of the aptamer and upon binding to the target, the structural reorganization causes the Gd^3+^-DOTA-labeled strand to dissociate from the duplex, which in turn increases the relaxation time and thus the brightness of the MRI signal [46]; (**B**) Vectoring to intended target: An aptamer is equipped with a contrast agent and will vector the probe directly to the intended target.

MRI is a highly efficient technique that provides non-invasive three-dimensional images of living systems and of biological events with sub-millimeter spatial resolution [37]. In MRI, exogenous contrast agents—mainly small molecules based on Gd^3+^-complexes—are used to enhance the image contrast by increasing the longitudinal (*T*_1_) or transverse (*T*_2_) relaxation times [38,39]. An important research avenue in the field of MRI consists in the development of smart or responsive contrast agents which consist either of systems that induce a change in magnetic relaxation in the presence of a biochemical stimuli (Figure 1A) or conjugates that vector MRI probes to their intended targets and sites (Figure 1B) [40]. Smart contrast agents based on aptamers have been devised by the application of both strategies. Indeed, in a proof-of-principle article, Yigit et al. developed a method for the detection and bisosensing of adenosine in vitro [41]. The contrast agent chosen in this system relied on biocompatible superparamagnetic iron oxide nanoparticles (SPIONs) due to their excellent capacity at changing the nuclear spin relaxation of neighbouring water protons [42]. The SPIONs were coated with cross-linked dextran which in turn could be functionalized with 3′- or 5′-thiol-modified DNA sequences that were designed so as to partially hybridize to a potent anti-adenosine aptamer [43]. In the presence of the adenosine analyte, the aptameric section refolded into its three-dimensional binding pocket concomitantly disrupting the hybridization to the SPION carrying oligonucleotides. The disruption of the SPION clusters led to the dispersion of single nanoparticles which display larger *T*_2_ values compared to the initial bioconjugate [41]. The observed brightening of the MR images was ascribed to the resulting increase in *T*_2_ values. In a related system, anti-thrombin aptamers [15,44] were immobilized on cross-linked dextran coated SPIONs and upon binding to the thrombin target, the nanoparticles assembled into larger aggregates which led to a decrease in *T*_2_ values (and thus a reduction of the brightness of MR images); a strategy that is often preferred in *T*_2_-weighted MR imaging [45]. More recently, a similar strategy was applied however by replacing the SPIONs with a Gd^3+^-based *T*_1_-weighted contrast agent [46]. Indeed, a Gd-DOTA complex was connected (by standard amide bond formation chemistry) to the 3′-amino modified end of a DNA oligonucleotide designed to be partially complementary to the adenosine aptamer. The aptameric part was connected to streptavidin and released the Gd-DOTA-modified oligonucleotide from the large streptavidin complex upon binding to the target adenosine. The release of the contrast agent bearing oligonucleotide in turn led to an increase in *T*_1_ value (and thus of the brightness of the MRI signal). In a conceptually related strategy, a catalytic DNA molecule (DNAzyme) [4,5] was used to release Gd-DOTA from a bulky complex [47]. Indeed, the RNA substrate was equipped with the Gd-DOTA complex while the UO_2_^2+^-dependent DNAzyme was connected to the protein streptavidin via a biotin moiety anchored at its 3′-end. In the presence of the analyte (UO_2_^2+^), the DNAzyme adopted its catalytically active structure and hydrolyzed the single embedded rA unit, releasing the contrast agent.

Monoclonal antibodies have been employed as tumor-specific ligands for the delivery of contrast agents [48,49]. By analogy to their proteinaceous counterparts, aptamers can play the role of vectors to transport contrast agents to specific regions of interest for MR imaging in vivo (Figure 1B) [50]. For instance, in a proof-of-principle study, the anti-thrombin aptamer was coupled to a Gd-DPTA (DPTA = diethylenetriaminepentaacetic acid) complex [51]. When the aptamer-Gd-DPTA bioconjugate was incubated with thrombin, significant relaxivity enhancements could be observed due to target interaction which increases the size of the contrast agent and concomitantly the rotational tumbling time [52]. In an ingenious system, Wang et al. bioconjugated a 2′-fluoropyrimidine-modified RNA aptamer specific for prostate cancer cells [53] on thermally cross-linked SPION [54]. The resulting construct not only allowed the transport of a contrast agent to the intended target and the concomitant MRI detection of prostate cancer cells in vitro but also served as a convenient scaffold for the selective delivery of the anticancer agent doxorubicin (DOX) [55]. The same RNA aptamer-SPION construct was then later used for the in vivo MRI detection of prostate tumors in a mouse model [56]. Related to this approach, a G-rich 26-nucleotide long aptamer coined AS1411 [57] was first conjugated to silver nanoclusters (Ag NCs) and then coupled to ultra-small gadolinium oxide (Gd_2_O_3_) nanoparticles [58]. The resulting Gd_2_O_3_-aptamer-Ag NCs system was successfully employed for the detection of MCF-7 tumor cells by MR and fluorescence imaging in vitro. Similarly, a variant of cell-SELEX was recently used to isolate aptamers that specifically bound inflamed human aortic endothelial (HAE) cells [59]. The resulting aptamer tightly bound the desired target (*K*_d_ = 82 and 460 nM for fixed and free HAE cells, respectively) and was conjugated to magnetic iron oxide particles for the efficient and selective in vitro detection of activated HAE cells.

### 2.2. Aptamers and PET Imaging

**Figure 2:**
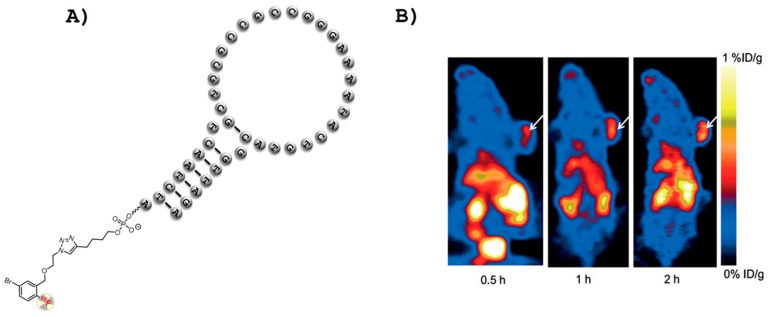
(**A**) Hypothetical secondary structure of the sgc8 aptamer and the ^18^F-label; and (**B**) positron emission tomography (PET) images of a mouse model with HCT116 tumors, white arrows represent the HCT116 xenograft [65].

Positron emission tomography (PET) is another highly accurate biomedical imaging modality that is used worldwide in clinical diagnostic applications due to its capacity at providing tomographic resolution at any tissue depth [60,61]. Several radioisotopes (e.g., ^18^F, ^64^Cu, ^11^C, ^13^N, ^124^I, and ^68^Ga) display suitable properties for PET, namely a decay by emission of a positively charged particle (the positron (β^+^)). Of these potential positron emitting radionuclides, ^18^F is often preferred due to its rather convenient half-life (*t*_1/2_ = 110 min), facile production, and favorable physical properties (clean decay and low emission energy) [62]. Besides the development of ^18^F-based synthons and radiolabeling strategies, an important challenge in the field of PET imaging is the crafting of target-specific imaging agents [61]. The potential of aptamers at delivering radionuclide probes was realized early on by Lange et al. who photoconjugated an ^18^F-labeled precursor on the 5′-amino-modified DNA thrombin aptamer [63]. More recently, an aptamer (sgc8) selective for the protein tyrosine kinase 7 (PTK7) [19] was ^18^F-radiolabeled and the resulting bioconjugate was used for the detection and the quantification by PET imaging of the expression of PTK7 both in vitro and in different tumor mouse models [64]. Similarly, the very same sgc8 aptamer was radiolabeled by application of the copper (I)-catalyzed alkyne-azide cycloaddition (CuAAC or click reaction) using a metabolically stable ^18^F-areene-arene derivative (Figure 2A) [65]. The affinity of the resulting ^18^F-sgc8 aptamer for the PTK7 target could be determined (*K*_d_ = 1.1 nM) by PET imaging in vivo and further used for the mapping of tumoral PTK7 expression (Figure 2B). ^18^F-arene tags were also connected by standard amide bond formation to an aptamer selective for the extracellular matrix glycoprotein tenascin-C which has been identified as a potential biomarker for various diseases, including myocarditis as well as different forms of cancer [66,67]. The anti-tenascin-C aptamer was also radiolabeled with a ^64^Cu-NOTA complex and both the ^64^Cu and ^18^F-labeled aptamers were used for the in vitro and in vivo PET imaging analysis of the stability of the aptameric construct and for tumor localization in a mouse model [67]. These first examples of in vivo PET imaging guided by aptamer ligands were followed by a recent article by Zhu et al. where DNA aptamers were screened both in vitro and in vivo against the cell membrane HER2 which is overexpressed in various types of cancer [68]. In a first step, a traditional in vitro selection experiment was carried out using a His-tagged extracellular domain of HER2 to isolate aptamers against this biomarker. Following eight rounds of the protein-based selection, seven rounds of cell-SELEX were applied with live SKOV3 ovarian cancer cells as targets to ensure proper binding of the aptamer candidates under in vivo-like conditions. This dual selection strategy allowed for the isolation of different high affinity (*K*_D_ values in the low nM range) aptamers against SKOV3 cells, which were subsequently ^18^F-radiolabeled by application of a click reaction protocol. The ^18^F-labeled aptamers were injected intravenously into an SKOV3 xenograft tumor and their tumor uptake efficiency was evaluated by PET imaging analysis. The most efficient radiolabeled aptamer was then successfully used for the PET imaging detection of HER2 in an ovarian cancer mouse model [68].

In an alternative methodology, a hybridization reaction between a radiolabeled sequence that is partially complementary to an aptamer can be used to circumvent the tedious and material consuming purification step involved in the direct labeling of an aptamer. In this context, using click chemistry, Park et al. ^18^F-radiolabeled an oligonucleotide that recognizes the anti-nucleolin aptamer AS1411 [57] and used the resulting duplex for the in vitro and in vivo PET imaging detection and targeting of C6 tumors in a mouse model [69]. As clearly shown in this section, the potential of aptamers to serve as PET imaging agents only begins to be explored and additional and alternative ^18^F-radiolabeling strategies [70,71,72,73] will certainly facilitate the application of aptamers in this imaging modality.

Aptamers have also been used in the related imaging technique SPECT (single photon emission computed tomography) where radionuclides (e.g., ^99m^Tc or ^111^In) decay by emitting a single γ-ray [74]. In a recent example, a 2′-fluoro-modified RNA aptamer (F3B) was raised against the human Matrix MetalloProtease-9 (hMMP-9) which is implicated in angiogenesis and believed to favor tumor cell formation [75]. The unmodified purine ribonucleotides were converted to 2′-*O*-methyl-modified units after SELEX and the resulting aptamer (F3Bomf) displayed a very high specificity and binding affinity for its intended hMMP-9 target (*K*_d_ = 20 nM). The fully modified aptamer F3Bomf could be connected to a ^99m^Tc complex and successfully used for the detection of the tumor biomarker hMMP-9 in human glioblastoma sections [75]. The same aptamer F3Bomf was subsequently radiolabelled with ^99m^Tc and ^111^In complexes which revealed to be excellent candidates for the in vivo detection of hMMP-9 in mice bearing human melanoma tumors [76].

## 3. Aptamers for the Treatment and Diagnostics of Neurological Diseases

Aptamers can prevent protein-protein interactions, protein aggregation, and inhibit enzymes and thus represent alluring biomolecules for the modulation and mechanistic investigation of biological events related to neurodegenerative diseases. Surprisingly, the use of aptamers in the field of neurosciences is rather modest but is steadily increasing since new perspectives of traversing the blood brain barrier are rising for those molecules [77,78,79].

### 3.1. Aptamers and Neurotransmitters

**Figure 3:**
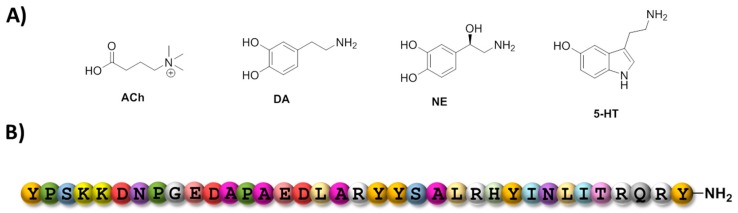
(**A**) Chemical structure of the main biogenic monoamine neurotransmitters; and (**B**) amino acid sequence of neuropeptide Y [96].

The transmission of signals between two neurons is relayed by the exocytotic release of a battery of distinct chemical entities called neurotransmitters (see Figure 3A). Neurotransmitters consist mainly of single amino acids and their metabolites (e.g., glutamate and GABA, respectively) [80], biogenic monoamines (e.g., dopamine (DA), norepinephrine (NE), acetylcholine (Ach), and serotonin (5-HT)) [81], soluble gases (mainly NO, CO, and H_2_S), and neuropeptides (e.g., neurokinin A and B, substance P or neuropeptide Y) [82]. In addition to their critical roles in numerous physiological functions, abnormal levels of neurotransmitters are indicators of various diseases including tumors [83], tauopathies [84], and psychological and mood disorders such as schizophrenia [82,85], However, due to the presence of only low amounts, complex and delicate matrix composition, and the inherent chemical nature of neurotransmitters, detection of variation of their local concentrations is a rather difficult undertaking, even on samples obtained by ex vivo preparation [81], Aptamers have already demonstrated their capacity at recognizing and sensing various neurotransmitters [86]. Indeed, in an early report, Mannironi et al. have isolated an RNA aptamer that specifically recognized dopamine (*K*_d_ = 1.6 µM for the free molecule in solution) [87]. This RNA aptamer was fundamental in the development of a potent dopamine biosensing system. This approach exploited the three-dimensional folding produced by the binding event which in turn favored gold nanoparticle aggregation leading to a colorimetric change [88]. Similarly, the RNA aptamer was used for the selective (despite the presence of competitive catecholamines) and sensitive (100 nM to 5 μM concentration range) electrochemical detection of DA [89]. Surprisingly, when the sequence of this anti-DA RNA aptamer was converted into its DNA counterpart, the affinity of the aptamer was increased and the specificity retained [90]. This DNA version of the DA aptamer was recently used in an in vivo study assessing its capacity at reversing cognitive deficits caused by the non-competitive NMDA-receptor antagonist, MK-801 in a rat model [91]. However, the specificity and binding capacity of the DNA homolog was seriously questioned recently, and the authors even suggested that it was not acting as a true aptamer [92].

Additionally, aptamers were also raised against the biogenic monoamines norepinephrine [93], acetylcholine [94], and serotonin (developed by Base Pair Biotechnologies, Inc., Pearland, TX, USA) [95].

Neuropeptides represent the largest family of neuromessengers and can modulate both gene expression and synaptic communication [82,97]. Due to their larger size and broader chemical diversity, neuropeptides bind to their targets with higher affinities than biogenic monoamines and are consequently present in even lower quantities. Neuropeptides thus represent attractive targets for aptamer selection to devise potent sensing and quantification systems. In this context, first selection campaigns aimed at raising aptamers against neuropeptide Y (Figure 3B), which is negatively charged (pI = 5.52) at pH 7.0, and thus represents a challenging target [98,99]. First, an RNA aptamer was isolated and shown to bind tightly to the C terminus of neuropeptide Y (*K*_d_ = 370 nM) and displayed no cross-reactivity with the closely related (~50% sequence homology) human pancreatic polypeptide (hPP) [99]. More recently, DNA aptamers were also raised against neuropeptide Y and displayed similar affinities (*K*_d_ values in the 0.3–1 µM range) and selectivities to the RNA counterpart [98]. One DNA aptamer was integrated in a graphene-gold nanocomposite-based sensing platform for the fast, selective, and precise in vitro detection of neuropeptide Y [100]. This sensing platform displays a detection limit of 10 pM as well as high selectivity and fast response. Similarly, Banerjee et al. developed an aptasensor based on carbon fiber amperometry to detect neuropeptide Y in pheochromocytoma 12 cells [101].

The undecapeptide substance P is a member of the tachykinin family and is an essential excitatory transmitter involved in numerous important biological activities and functions. In light of its high biological and neurological significance, an RNA aptamer was isolated against substance P [102]. Indeed, an automated SELEX procedure with the D-peptide of substance P as target was applied to isolate an L-RNA aptamer which could be converted to its corresponding Spiegelmer (D-RNA) [103] which bound to the naturally occurring L-substance P with high affinity (*K*_d_ = 40 nM). This Spiegelmer was also efficiently used to inhibit the substance P-mediated calcium release in human AR42J cells (IC_50_ = 45 nM). A similar strategy was applied in the isolation of a Spieglemer (D-RNA) aptamer against the neuropeptide nociceptin/orphanin FQ (N/OFQ), involved in numerous capital biological and neurological responses such as anxiety, pain, and stress [104]. The most potent Spiegelmer, NOX 2149, recognized N/OFQ with high affinity (*K*_d_ = 0.2 µM) and concomitantly prevented N/OFQ from binding to its receptor (IC_50_ = 110 nM). Aptamers have also been raised against the neuropeptides somatostatin [105], ghrelin [106], Glucagon [107], angiotensin II [108], and calcitonin gene-related peptide 1 (α-CGRP) [109], as well as against certain receptors such as neurotensin receptors [110,111], and the cholecystokinin B receptor [112].

### 3.2. Aptamers and Tauopathies

Tauopathies are progressive neurodegenerative disorders including Alzheimer’s disease (AD), Parkinson’s disease (PD), Huntington’s and prion diseases, and are characterized by the presence of aggregates of the microtubule-associated protein tau in the brain [113]. Even though the exact origins and the molecular mechanisms are vastly unknown, it is believed that misfolded and abnormal forms (often hyperphosphorylated) of the wild-type proteins are involved in the physiopathology of these diseases by acting as seeds for the aggregation of these proteins. Aptamers could thus contribute to this field as tools for the investigation of the origin of tauopathies and for the detection, the prevention, and the treatment of these disorders [77,78], as highlighted in this section for AD and PD.

#### 3.2.1. Alzheimer’s Disease

In AD, the combined accumulation and deposition of abnormal forms of tau protein and amyloid β (Aβ) peptides in the human brain is followed by a progressive functional disruption of neuronal networks [114]. Consequently, both tau and Aβ proteins represent valid targets for aptamer selection experiments.

Tau proteins play an important role in the stabilization and assembly of microtubules and display little propensity at aggregating and oligomerizing when found in their native folds and states. In AD, aggregates of hyperphosphorylated tau are thought to be transmitted in a prion-like manner that proceeds along connected neurons throughout the brain (the so-called tau-hypothesis) [115]. The understanding of how and why tau protein aggregates are capable of propagating in the brain is an important issue in neurosciences. In order to develop new tools to investigate and prevent this aggregation, Kim et al. have recently reported the isolation of an RNA aptamer against the longest isoform of human tau (tau40, 2N4R) [116]. The resulting aptamer efficiently prevented the oligomerization of tau monomers in vitro without affecting its degradation but on the other hand was not capable of disentangling pre-existing tau oligomers. Interestingly, the RNA aptamer could also delay tau oligomerization in DOX-inducible tau HEK293 cells and significantly reduced interneuronal tau propagation in primary rat neuronal cells, underscoring the inhibitory potential of aptamers for the regulation of tau oligomerization. A ssDNA sequence capable of binding to the human tau isoforms 381 and 410 (*K*_d_ = 0.19 and 0.35 µM, respectively) was identified not by SELEX but by kinetic capillary electrophoresis [117]. This DNA oligonucleotide was subsequently used in an aptamer-antibody sandwich assay for the detection of tau 381 in human plasma (limit of detection of 10 fM) [118].

**Figure 4:**
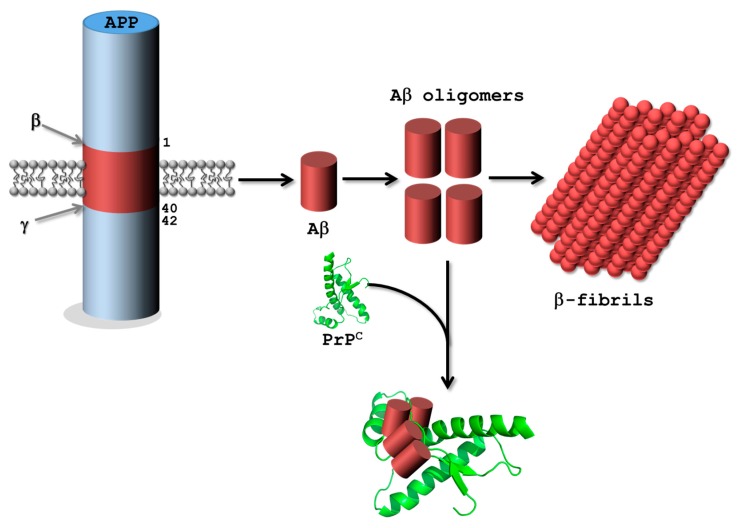
Schematic depiction of the formation of Aβ fibrils: BACE1 cleaves APP (Aβ sequence numbers are shown only) in the extracellular domain and the resulting fragment remains membrane-bound where it is cleaved by the γ-secretase complex into Aβ peptides (only the main Aβ40 and Aβ42 isoforms are shown). Monomeric Aβ peptides then aggregate to form oligomers and eventually β-fibrils. The cellular prion protein PrP^C^ can also bind to Aβ oligomers [78,119].

In addition to abnormal tau protein accumulation, the deposition of rather short (~4 kDa) Aβ peptides is believed to be a key step in the progression of AD—known as the Aβ-hypothesis [119]. Aβ peptides stem from the sequential cleavage of the larger transmembrane glycoprotein amyloid precursor protein (APP) mainly mediated by the combined action of a β-secretase (also known as β-site APP cleaving enzyme-1; BACE1) and a γ-secretase complex (Figure 4). Both aspartyl proteases hydrolyze APP into several Aβ isoforms (mainly Aβ38, Aβ40, and Aβ42) which first assemble into synaptotoxic oligomers and then into amyloid fibrils, often considered as one of the major toxic agents in AD [119]. Lastly, Aβ oligomers also seem capable of binding to normal prion protein (PrP^C^) with high affinity which might be at the origin of the toxicity of these oligomers [120]; this hypothesis, however, remains somewhat controversial [121,122,123]. Consequently, PrP^C^, Aβ monomers and oligomers, BACE1, and the γ-secretase complex all represent relevant targets for aptamer selections to modulate, inhibit, or understand their functions [78,79].

Inhibition of the catalytic activity of BACE1 or the γ-secretase complex could directly hinder the formation of the toxic Aβ oligomers and β-fibrils. Consequently, both a natural and a modified DNA aptamer have been raised against the transmembrane protease BACE1 [124]. The natural DNA aptamer binds BACE1 with high affinity (*K*_d_ = 69 nM) and was shown by a FRET assay to inhibit its activity both in vitro (IC_50_ = 242 nM) and in an AD cell model [124]. The modified aptamers, selected with the triphosphate 5-chloro-dUTP and 7-deaza-dATP [125], displayed similar properties to the unmodified sequence, albeit with a slightly higher binding affinity and an interesting agonist/antagonist behavior [126]. Similarly, an RNA aptamer (called S10) was selected against the cytoplasmic tail B1-CT of BACE1 and showed a remarkable affinity for both phosphorylated and nonphosphorylated BACE1 (*K*_d_ = 330 and 360 nM, respectively) [127]. Aptamer S10 was also capable of binding to cellular BACE1 and did not prevent binding of the protein factor GGA1 to the cytoplasmic tail or the casein kinase-mediated phosphorylation of the single serine S498 located in the B1-CT tail. Thus far, no aptamers have been raised against the membrane bound γ-secretase nor any of its constitutive proteins (i.e., presenilins, nicastrin, APH-1, and PEN-2) [119].

The Aβ40 and Aβ42 isoforms readily associate to form soluble but toxic Aβ oligomers. In this context, an RNA aptamer coined β55 was identified by SELEX and shown to recognize its intended target Aβ40 with high affinity (*K*_d_ = 29 nM), albeit not in its monomeric or oligomeric forms but as fibrillar assemblies [128]. Furthermore, Farrar et al. also demonstrated that β55 was capable of binding to amyloid plaques in ex vivo human AD brain tissue slices. Remarkably, this binding event was also confirmed in vivo using a fluorescently labeled β55 in a transgenic mouse model [129]. Having realized that β55 only bound to Aβ40 polymers despite using a monomeric species in SELEX, Rahimi et al. set up an in vitro selection experiment that involved covalently-stabilized oligomers of Aβ40 [130]. Surprisingly, the resulting RNA aptamers did not bind to Aβ40 oligomers but only to Aβ40 fibrils, showing the high and natural propensity of nucleic acids to recognize fibrillar motifs in protein assemblies. This propensity was further confirmed by the substantial cross-reactivity of the isolated aptamers with Aβ42 and other amyloid fibrils. Another set of RNA aptamers was obtained through a selection experiment that used monomeric Aβ40 conjugated to gold nanoparticles; this target was hypothesized to act as a model of Aβ oligomerization and to allow both binding to the Aβ40 peptide and facile separation from unbound material [131]. Two aptamers, obtained by different elution protocols from the Aβ40-gold nanoparticles, indeed recognized monomeric Aβ40 (*K*_d_ values of 22 and 11 μM) and were capable of inhibiting Aβ fibrilization.

Lastly, the prion protein (native PrP^C^ or infectious isoform PrP^Sc^) is an interesting target for aptamer selection due to its implication in AD (Figure 4) and prion diseases such as spongiform encephalopathies. Consequently, various DNA and RNA aptamers have been isolated against bovine [132], mouse [133], and human [134] PrP^C^ as well as PrP^Sc^ [135]. However, we refer the interested reader to a recent and concise review article thoroughly covering this topic [77].

#### 3.2.2. Parkinson’s Disease

As for AD, the origin of the PD pathology is believed to be connected to the formation of misfolded protein aggregates. However, in PD, the presynaptic neuronal, 140 amino acid-long protein α-synuclein and not Aβ has been identified as a possibly responsible agent for the pathogenesis [136]. The aggregation of α-synuclein proteins into oligomers eventually leads to the formation of fibrils which then accumulate in cytosolic filamentous inclusions called Lewy bodies, which are the hallmark of PD [137]. Consequently, the first example of a DNA aptamer against α-synuclein—coined M5-15—was obtained by SELEX using the monomeric protein as target in the selection protocol [138]. However, M5-15 preferentially bound to α-synuclein oligomers and did not recognize the monomeric form. This inherent conformation specificity prompted the authors to isolate other DNA aptamers using a competitive screening method based on aptamer blotting and α-synuclein oligomers as target [139,140]. The resulting aptamers presented *K*_d_ values in the low nM range that selectively recognized the oligomeric form of α-synuclein over monomers and fibrils [140]. Interestingly, the isolated aptamers also bound to Aβ40 oligomers (with slightly lower *K*_d_ values), hinting at the possibility of a selective recognition of aggregates with β-sheet-rich proteins. One of the isolated aptamers (T-SO517) was recently integrated in a potent label-free aptasensor system for the selective detection of α-synuclein oligomers (the limit of detection was in the low nM or pM depending on the analysis method) [141]. Aptamer T-SO517 was also integrated in a fluorescent sensing platform that enabled the detection of Aβ40 oligomers down to 12.5 nM [142]. Similarly, a nanocomposite of aptamer T-SO508, gold nanoparticles, and thionine was used as probe for the selective and sensitive detection of Aβ40 oligomers (100 pM limit of detection) [143]. Lastly, variants of T-SO508 were either grafted on magnetic nanoparticles to detect Aβ oligomers (detection limit of 36 pM) [144] or combined with abasic site-containing molecular beacons to monitor Aβ aggregation [145].

Lastly, low levels of dopamine are also frequently found in patients suffering from PD and thus, all the anti-DA aptamers described in Section 3.1 could be of use for the detection, monitoring, and treatment of PD. Other potential targets are the other two members of the synuclein family, namely β-synuclein and γ-synuclein which might be involved in neurodegenerative diseases.

## 4. Aptamers as Key Components in the Fabrication of Smart DNA Origami Objects

The predictable nature and the high degree of fidelity of DNA base pairing are the origin of the development of a plethora of DNA-based nanomaterials [3,146]. Of particular interest are DNA origamis which are created by combining large single-stranded DNA frameworks (e.g., M13 bacteriophage genomic DNA (7249 nucleotides) [147]) with hundreds of shorter (20–60 nucleotides) oligonucleotides (called staple strands) partially complementary to particular sequences on the genomic DNA scaffold so as to form pre-designed two- and/or three-dimensional folds [3,148]. Since both aptamers and DNA origami are made out of the very same biopolymer and because of the inherent binding capacity of aptamers, a combination of both nucleic acid-based materials has been used for different applications including: (1) defined spatial positioning of proteins (and other ligands of interest) on DNA arrays for medical diagnostic, biosensing, or tissue and material engineering purposes [149,150]; (2) development of nanorobots for the delivery of drugs and other payloads [151,152]; and (3) the creation of multimodal sensing platforms [153].

**Figure 5:**
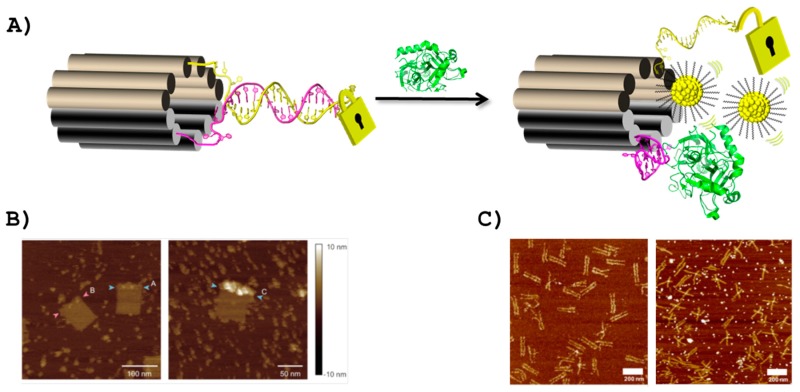
(**A**) Schematic representation of the aptamer-gated DNA nanorobot [151]: the aptamer (magenta) is locked in a double-stranded form by a partially complementary sequence (yellow) and both are grafted on the nanorobot. The payloads (either gold nanoparticles (shown) or antibody fragments) are constrained to remain within the DNA construct and only the recognition of intended target by the aptamer (green) will unlock the DNA nanorobot and enable the delivery of the payload; (**B**) AFM images of a DNA origami-aptamer construct in the presence of a non-target protein (hLDH; left-hand side) and presence of the target protein (*Pf*LDH; right-hand side) [152]; (**C**) AFM analysis of a DNA origami equipped with a split aptamer system in the closed (left-hand side) and open (right-hand side) forms [153].

Chhabra et al. were the first to report on the immobilization of proteins at specific locations of two-dimensional DNA nanoarrays [149]: two tile double-crossed (DX) DNA molecules [154] were equipped either with the thrombin aptamer [44] or an aptamer raised against the Platelet-derived growth factor (PDGF) [155] and combined with another set of DX tiles to form a two-dimensional DNA network with interspaced alternating lines of both aptamers. An atomic force microscopy (AFM) analysis showed that the corresponding target proteins successfully bound to their respective aptamers following their sequential addition on the network. This approach was then extended to a DNA origami constructed with 200 staple strands which resulted in the formation of rectangular two-dimensional DNA nanoarrays. The inclusion of the two aforementioned aptamers controlled the spatial positioning of the respective targets (i.e., thrombin and PDGF proteins) and therefore the generation of programmable high-density protein-DNA nanoarrays [149]. In a conceptually related construct, two aptamers binding to different epitopes of thrombin [15,44] were appended at different locations of rigid four- or five-helix bundle DNA tiles to evaluate the optimal inter-aptamer distance that ensures the highest binding to thrombin after formation of the hetero-aptamer system [150]. The optimal inter-aptamer distance appears to be at around 5.3 nM (i.e., slightly over the size of thrombin) for high affinity bivalent binding (*K*_d_ ~ 10 nM). The same strategy was then applied for the construction of a rectangular-shaped two-dimensional DNA origami object [156] two lines of each aptamer separated by 5.3 nM were included in the scaffold of the origami tile resulting in efficient bivalent binding to thrombin by dual-aptamer lines. These initial proof-of-principle studies paved the way for the development of smart DNA origami objects. For instance, in a landmark work, Douglas and co-workers designed a DNA nanorobot in the form of a hexagonal barrel (Figure 5A) consisting of 196 staple oligonucleotides and the 7308 nucleotide-long genomic DNA of an M13-like phage [151]. The two domains that constitute the barrel are connected by single-stranded oligonucleotidic hinges at the rear and closed in the front by an aptamer sequence hybridized to a partially complementary sequence. In the presence of protein tyrosine kinase 7 (PTK7), the sgc8c aptamers (specific for PTK7) [19] dissociate from the lock-duplexes and the DNA nanorobot opens as a direct consequence of the formation of the aptamer-ligand complex. The different payloads (i.e., either 5-nm gold nanoparticles or antibody fragments against the human leukocyte antigen–A/B/C) are affixed on the inner walls of the barrel (either through 5′-thiol or 5′-amino modified linkers, respectively) and can be released upon opening of the DNA nanorobot. This ingenious system was incubated with different cell lines expressing the HLA–A/B/C antigen with a combination of molecular inputs. An increase in fluorescence caused by the binding of the antibody fragments on the cell surface could only be detected in the presence of the correct “key”, namely PTK7. Lastly, the DNA origami system could be extended to yield a more discriminatory system by incorporating combinations of aptamers recognizing different targets.

Godonoga et al. recently reported a DNA origami-aptamer construct for the specific recognition of a malaria biomarker [152]. In this system, a rectangular DNA origami [156] was fabricated by including the aptamer 2008s that specifically recognizes the malaria biomarker *Plasmodium falciparum* lactate dehydrogenase (*Pf*LDH) with high affinity (*K*_d_ = 42 nM) through the formation of a 2:1 aptamer:ligand complex [157]. An AFM analysis clearly revealed that only *Pf*LDH and not the related human homolog (hLDH) bound to the surface of the DNA origami decorated with the aptamer 2008s (Figure 5B), thus clearly demonstrating that large supramolecular DNA constructs could be used as diagnostic tools for diseases.

Lastly, DNA origami-aptamers systems can also be used as smart biosensing platforms. In a recent contribution, Walter et al. blended the pinching capacity of a nanomechanical DNA origami forceps [158] with the biosensing capacity of split aptamer systems [159]. Indeed, the constituting sequences of the ATP-specific split aptamer were equipped with green- and red-emitting photostable cyanine dyes that act as energy donor and acceptor, respectively [160] and were subsequently appended on one arm of the DNA origami forceps. In the absence of the analyte (i.e., ATP) the constructs remained in an open form (Figure 5C) and emitted green light (absence of energy transfer). On the other hand, in the presence of ATP, the split aptamer sequences bound together which resulted in the closure of the DNA origami forceps which concomitantly led to red light emission due to an efficient energy transfer (Figure 5C). The sensing event can be observed by both AFM (Figure 5C) or by the change in fluorescence from green to red, hence following the concept of DNA traffic lights [161].

## 5. Aptamers as Drug Carriers and Gene Regulating Agents

Repair of the biological damage or chemical imbalance caused by a disease often requires an intervention in the form of a drug at the precise location of the inflicted disorder. Depending on the disease, the nature and the location of the damaged biomaterial can vary substantially ranging from simpler constructs such as proteins or nucleic acids to larger systems such as cells or entire tissues. In order to improve the therapeutic index of drugs and reduce offside effects and some inherent toxicity, numerous systems have been devised to carry drugs in a stable form to the intended sites [162,163]. In this context, aptamers represent ideal candidates as delivery systems for conventional or encapsulated drugs, but also for therapeutic oligonucleotides and peptides due to their properties (vide supra) [25].

### 5.1. Nanomaterials Conjugated Aptamers

Due to the ease of functionalization of oligonucleotides, aptamers can be conjugated to virtually any type of nanomaterial [164]. Amongst these, graphene oxide (GO) holds high promises for the development of advanced materials due to its interesting optical and electronic properties but also because of the possibility of constructing barrier films and new classes of membranes. In addition, graphene oxide consists of interspaced sheets that form 2D structures which are known to exhibit low toxicity, high mechanical flexibility, a large accessible surface, and an excellent quenching capacity of fluorophores [165]. Consequently, numerous reports exist on the crafting of GO-aptamers conjugates, especially for the development of biosensors [166]. In a highly interesting approach, Nellore et al. explored the potential of immobilizing aptamers selective for biomarkers on GO to capture and identify circulating tumor cells (CTCs) in blood—an approach that is reminiscent of that described earlier in this review for the immobilization of aptamers on DNA origamis (see Section 4) [167]. Thus, aptamers selective for the prostate-specific membrane antigen (PSMA), HER2, and the carcinoembryonic antigen (CEA) biomarkers were covalently affixed on two-dimensional GO sheets by standard amide bond formation with the carboxylic acid residues present on GO and then converted to a three-dimensional architecture using polyethyleneglycol (PEG) as a cross-linking agent. The high capacity of the resulting aptamer-coated 3D GO foam-based membrane at capturing tumor cells was proven by separating the different cells from infected rabbit blood. In a related study, Bahreyni et al. connected aptamers selective against the membrane protein mucin MUC1 which is overexpressed in various epithelial carcinomas, through π-π stacking interactions with the GO surface [168]. GO was charged either with the anti-MUC1 aptamer or a fluorescently-labeled aptamer selective for MUC1 cytochrome C. Upon successful internalization of the labeled aptamer nano complex into target MDA-MB-231 and MCF-7 cells, a strong fluorescent signal was observed indicating binding and release of the fluorescein labeled aptamers to cytochrome C, while no effects were observed with non-targeted cell lines (HepG2). This theranostic system appeared to be non-invasive and selective for the targeted cells inducing apoptosis.

An elegant “on and off” strategy reported by Tang et al. required a non-covalent assembly of the Cy5.5-labeled AS1411 aptamer, targeting nucleolin, on the surface of a GO-wrapped, DOX-loaded mesoporous silica nanoparticle [169]. This system allowed a light induced administration of DOX that could be monitored by fluorometric measurements: a first fluorescent signal of the Cy5.5-labeled aptamer indicated real time endocytosis of the aptamers into the target cell, while a second fluorescent signal was induced by laser irradiation because absorption and transduction of the near infrared light by the GO structures lead to local heat and expansion of the GO sheets and thus the release of the DOX molecules in a light dependent manner.

Besides GO, gold-based nanomaterials and derivatives have attracted considerable attention for the crafting of aptamer-based materials and devices due to their stability and their advantageous optical and electronic properties [170,171,172]. In particular, gold nanoparticles have served as drug delivery systems for aptamers since they generally increase the stability of ssDNA towards nuclease degradation, the cellular uptake capacity of oligonucleotides, as well as their biocompatibility and usually induce a limited immune response [173,174]. For instance, Huang and coworkers used gold nanoparticles to attach thrombin-binding aptamers on their surface [175]. The complexed thrombin on the gold nanoparticles (d = 13 nM) efficiently inhibited the thrombin activity against fibrinogen upon activation with green laser light. The anticoagulant activity of these complexes was found to be 30 times more potent than recent commercially available drugs (heparin, argatroban, hirudin, or warfarin) exhibiting a good biocompatibility, low toxicity and showed excellent half-life stability in serum (*t*_1/2_ >14 d) [175].

Besides the use of gold nanoparticles as drug delivery systems and diagnostic tools, Niu et al. demonstrated that conjugation of the sgc8c aptamer [19] with a gold *N*-heterocyclic gold (I) complex (NHC-Au^I^-aptamer), known to induce cell apoptosis, resulted in internalization of the bioconjugate specifically into CCRF-CEM leukemia cells and exhibited excellent cytotoxicity [171]. The sgc8c-7aptamer employed in this strategy targeted the receptor protein tyrosine kinase 7 (PTK-7) that is overexpressed in CCRF-CEM leukemia cells and allowed for a 30-fold increase in cytotoxicity compared to the unmodified NHC-Au^I^ complex. No toxicity to off-target cells (in this case, K526 cells) was observed with the bioconjugate, on the other hand, a dose-dependent toxicity was observed with CCRF-CEM cells, underscoring the usefulness of this approach.

Quantum dots (QDs) represent another very attractive class of inorganic nanoparticles for the formation of aptamer bioconjugates [176,177,178]. QDs have unique photophysical properties, including color tunability and bright and extremely photostable fluorescence, that is keeping them in the forefront of numerous sensing applications [178]. In addition, QDs have also been used in dual imaging-drug delivery systems based on aptamers [179]. For instance, Su and co-workers covalently attached a capture sequence on a near infrared CuInS_2_-QD via amide coupling to connect a MUC1-aptamer to this nanoparticle through the formation of Watson–Crick base pairs [180]. Multiple CG-motifs present in the resulting duplex served as intercalation sites for daunorubicin (DNR), which is a drug for the treatment of acute myeloid leukemia. This system allowed a specific delivery of the DNR to prostate cancer cells in vitro and a concomitant sensing of the presence of DNR in the construct due to a marked drop in fluorescence intensity upon binding of the drug. A high cytotoxicity was found for MUC1 positive PC-3M cells but not for MUC1 negative HepG2 cells when treated with the DNR-loaded bioconjugate [180].

In addition to inorganic frameworks, aptamers can be coupled to various organic nanomaterials including DNA constructs [164], DNA micelles [181,182], aptamer-based hydrogels [183], lipids [184], or even vitamins [185,186]. Recently, Dai et al. developed a DNA tetrahedron (Td) labeled with an aptamer targeting MUC1. In this drug delivery system, the aptamer serves for the targeted delivery to MUC1-positive breast cancer cells while the DNA tetrahedron is instrumental for the intercalation of DOX [187]. A fluorescence-based drug loading experiment showed that each aptamer-Td construct with an average size of 12.4 nM could carry up to 25 DOX molecules. A high red fluorescent signal of free DOX molecules could be observed upon binding of the negatively charged complex to the MUC1 positive breast cancer cells but not with MUC1 negative cells. Accordingly, a very high cytotoxicity was observed when MUC1 positive cancer cells were treated with the aptamer-Td conjugate [187].

A last category of nanoparticles that will be considered consist of linear block copolymers and dendritic polymers which have been extensively used as encapsulating devices in drug delivery systems [188,189]. Aptamers have also been used to decorate the surface of these polymeric entities and to facilitate targeted delivery. This concept was developed by the Langer laboratory who reported a strategy for a drug encapsulation by a triblock copolymer comprising a controlled release polymer (poly (lactic-co-glycolic-acid); PLGA), a hydrophilic polymer (PEG), and the 2′-fluoro-modified RNA A10 aptamer (see Section 5.5 and Section 6) that selectively recognizes the prostate cancer specific membrane antigene (PSMA) [55,190]. By systematically changing the composition of the complex, a narrow ratio between PEG and aptamer could be determined that enabled maximal specific binding to the target and cellular uptake, efficient drug (in this case the anticancer drug Docetaxel) release, high antibiofouling properties, and minimized self-aggregation and thus undesired accumulation in the spleen [190]. A similar approach was also used to selectively deliver a Pt(IV)-prodrug [191], a cisplatin prodrug [192], and other anticancer drugs [193,194,195], while dendritic polymers-aptamer systems have also successfully been used for in vivo [196] and in vitro [197] tumor imaging and drug delivery [198].

### 5.2. Micelles and Liposomes Conjugated Aptamers

Liposomes are small spherical and artificial vesicles with one or multiple lipid bilayers and have already been suggested as potentially highly efficient drug deliver systems at an early stage [199]. Indeed, Huwyler and co-workers already described in the early 1990s the use of liposomes decorated with monoclonal antibodies to delivery encapsulated tritium-radiolabeled daunomycin in a directed manner to target cells [200]. This strategy has many benefits including the modulation of the affinity of the bionconjugate complex by simply changing the ratio of antibodies present on the lipid bilayer surface along with the impressive quantity of drug molecules (>10,000) that can be delivered in a selective manner [200,201]. In a more recent study, Ara et al. used liposomes composed of lipid bilayers that are labeled with an aptamer instead of a monoclonal antibody [202] to ensure selective cellular uptake [203]. The aptamers were covalently linked on PEG 2000 disteraoyl phosphotheanolamine and targeted the primary cultured mouse tumor endothelial cells (mTEC). By application of a standard lipid hydration method, the corresponding aptamer-labeled PEG2000 liposomes and PEG200 liposomes (negative control) were formed. Fluorescent measurements and confocal laser scanning microscopy showed an increased uptake in mTEX cells of the aptamer-PEG liposomes compared to the unlabeled liposomes. In vitro experiments revealed that approximately 39% of the aptamer-PEG-liposomes could escape the endosomes in a receptor mediated way followed by clathrin-mediated endocytosis. Lastly, in vivo experiments with the aptamer-conjugated liposomes were performed with human renal cell carcinoma (OS-RC-2 cells) inoculating mice using confocal laser scanning microscopy. These experiments clearly indicated that the aptamer modified liposomes strongly accumulated (co-localization ratio of 25%) on tumor vasculature compared to non-labeled liposomes, where a co-localization ratio of only 3% accumulation was observed [203]. In a similar approach, a 2′-fluoro-modified RNA aptamer against the cancer stem cells (CSC) surface markers CD44 was affixed on the surface of a PEG-functionalized liposome by application of the thiol-maleimide click reaction [204]. The resulting construct was shown to act as a very efficient potential drug delivery system due to its high selectivity and specificity for CD44 positive cells.

**Figure 6:**
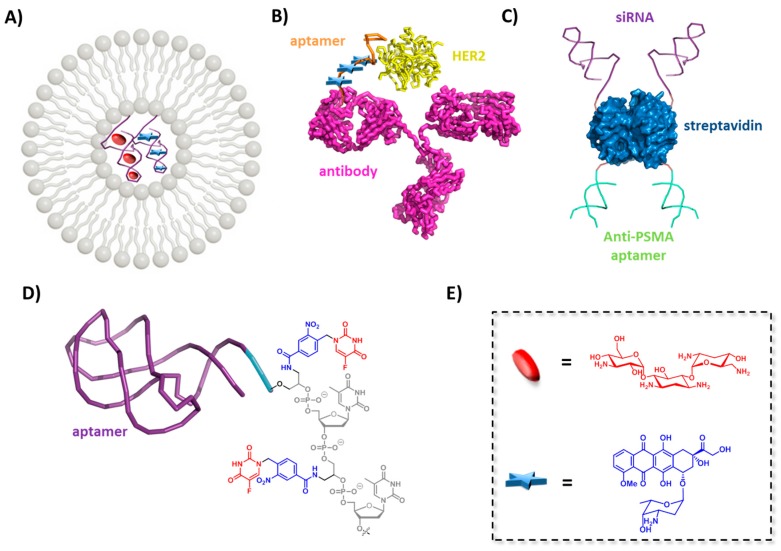
Illustrative examples of aptameric-based systems used as drug delivery systems: (**A**) encapsulation of aptamer-drug complexes in liposome [205]; (**B**) antibody-aptamer pincers (AAPs) for selective targeting of HER2 and delivery of DOX [208]; (**C**) streptavidin serves as the core for the connection of two anti-PSMA aptamers and two siRNA molecules [209]; (**D**) delivery of the 5-fluorouracil (5-FU; shown in red) drug connected to an aptamer-oligonucleotide scaffold via a photo-cleavable linker (shown in blue) [210]; and (**E**) chemical structures of tobramycin (red) and DOX (blue).

In a recent study by Plourde et al., the high binding capacity of aptamers served as a driving force for the incorporation of DOX into cationic liposomes (Figure 6A) [205]. Indeed, different versions of an anti-DOX DNA aptamer [206] were designed by either adding an additional base-pair on the binding motif or by including two binding motifs into one construct. Upon intercalation of DOX into the aptamer its intrinsic fluorescence was quenched and was used to determine both the loading efficiency and the binding affinity. The drug aptamer complexes were incorporated into cationic liposomes (d < 200 nM) via electrostatic interactions, where encapsulation of DOX into liposomes was greatly enhanced (ten times higher) when using an aptamer compared to negative controls without any specific DOX binding sites. This aptameric-encapsulation was compared to the Doxil-like formulation, which is a commercialized version of liposomes of DOX that allows the loading of up to 10,000 molecules through strong entrapment but concomitantly reduces the therapeutic efficiency due to a slow release [207]. The aptamer-based loading strategy offered a number of advantages over the Doxil-like formulation including similar loading capacity, faster release, and a higher therapeutic efficiency. Cytotoxicity assays revealed that liposomes with aptamers having an intermediate affinity (*K*_d_ = 334 nM) for DOX exhibited the higher therapeutic effects. The same approach was then extended to the amphiphilic drug tobramycin used for the treatment of lung infections. It is known that encapsulation of tobramycin in liposomes increases the in vivo efficacy compared to the free drug. The low encapsulation efficiency of tobramycin in liposomes is a limiting factor in this approach. The active loading with an aptamer allowed the encapsulation of up to six times more drug compared to passive encapsulation. Therefore, the authors reasoned that this strategy could find a broad application to load a large variety of drugs into liposomes.

### 5.3. Aptamer-Drug Conjugates

Aptamers have also been considered to guide drugs directly and selectively to their intended targets by direct conjugation to the therapeutic agent or through a small linker arm [211]. An early example of a covalent attachment of a drug on an aptamer is the appendage of DOX on the sgc8c-aptamer via a short linker. Indeed, the linker was connected to the aptamer by means of the thiol-ene click reaction and to DOX via a hydrazone moiety. The hydrazone unit was chosen because of the compatibility with the ketone group found on DOX and a possible hydrolysis in the acidic environment (pH 4.5–5.5) of endosomes [212]. In vitro tests showed that the administration of unconjugated DOX exhibited a higher toxicity towards non-targeted cells compared to the aptamer-DOX conjugate.

In an attempt to overcome low aptamers-drug ratios, Wang et al. synthesized an aptamer containing multiple copies of 5-fluorouracil (5-FU), a drug against colorectal and pancreatic cancer, via a photo-cleavable linker in order to spatially and temporarily control the drug release (Figure 6D) [210]. Besides covalent attachment of drugs on aptamers, a few examples of non-covalently attached drug-aptamer conjugates have also been reported, mainly by intercalation. The benefit of this strategy stems from the steady increase in loading capacity of the intercalating drug on aptamers with aptamer vs. drug ratios that improved from 1/1.2 in 2006 [55] to nearly 1/50 in 2013 by the use of self-assembling dsDNA drug intercalation sites directly appended to the desired aptamers [213].

Most drug delivery systems rely on the presence of a single type of aptamer which results in an efficient escorting of drugs to the intended target. However, since aptamers are usually obtained by in vitro selection experiments, small conformational changes of their targets caused by their in vivo environments might interfere or even impede their binding activities [68,214,215]. A multimeric aptamer-drug delivery strategy was developed in order to face this potential drop in binding affinity. The system consists of two distinct aptamers specific for different cancer subtypes and DOX intercalated in the double-stranded portions created by the self-assembly of the two aptameric species [216].

### 5.4. Aptamer-Antibody Conjugate

The conjugation of aptamers with their proteinaceous counterpart antibodies is used to increase the affinity of the resulting construct for a single target [7]. The rationale behind this strategy is based on the observation that antibody dimerization causes a decrease in *K*_d_ value (caused by lower *k*_off_ and higher *k*_on_ rates) compared to the two individual antibodies [208,217]. This working hypothesis was validated by an inhibition study of the VEGF-A and PDGF-B signaling pathways [218] as well as for the fluorescence detection of human CD4 [217]. More recently, Kang and Hah reported a drug delivery strategy based on the formation of an antibody-aptamer hybrid complex in order to improve the specificity of the construct for thrombin or the anti-human epidermal growth factor 2 (HER2) as model systems (Figure 6B). The resulting so-called antibody-aptamer pincers (AAPs) were found to increase the affinity for thrombin of the conjugate compared to the individual aptamers or antibody by 35-fold or 100-fold, respectively (*K*_d_ value of 567 pM). The DOX loaded AAP constructed with the anti-HER2 aptamer and antibody was also found to exhibit a 3–6 fold higher cytotoxicity than the individual antibody DOX conjugate or unconjugated DOX [208].

The conjugation of an aptamer with an antibody (a so-called oligobody) can also be helpful to overcome both the poor pharmacokinetics for systemic administration of the small aptamers and the limited tissue penetration of the rather large antibodies (~150 kDa) [219]. The combination of an anti-cotinine antibody (cot-body) with a cotinine labeled vascular endothelial growth factor (VEGF) targeting aptamer (cot-pega) led to the formation of an oligobody that exhibited no loss in affinity to cancer cells compared to the aptamer only, penetrated deep into tumor tissue of an A549-xenograft mouse model, displayed extended half-life times in serum (*t*_1/2_ = 8.3 h) and reduced tumor growth. All these studies clearly demonstrate the potential of aptamer-antibody conjugates in anticancer therapeutics.

### 5.5. Aptamers and Gene Regulating Agents

Aptamers, being of nucleic acid nature, can easily be conjugated to relevant RNA or DNA sequences (for instance to sgRNA in the gene editing system CRISPR/Cas9 [220,221]) and particularly to therapeutic oligonucleotides (siRNA, miRNA, or antisense agents) [222,223,224].

The first siRNA-aptamer conjugate was reported by McNamara et al. who tried to improve the therapeutic efficacy of siRNAs by constructing a chimera with aptamers as vectors to achieve targeted delivery [225]. The aptamer-siRNA chimera was constructed to target the cell surface receptor prostate specific membrane antigen (PSMA) overexpressed in prostate cancer cells via the A10 RNA aptamer [53], while the delivered siRNA targeted the polo-like kinase 1 (PLK1) and B-cell lymphoma 2 (BCL2) genes, which are overexpressed in numerous human tumors [225]. Upon internalization and processing of the RNAs by Dicer, the siRNA is directed to the RNAi pathway and silence their cognate mRNAs which in turn leads to the depletion of the PLK1 and BCL2 survival genes and ultimately cell death. The benefit of that system compared to chimeras of siRNA with antibodies is the low immunogenicity of the RNA aptamers and also an increased in vivo tissue penetration due to the small size. Additionally, it was demonstrated that the genes were only regulated in cells expressing PSMAs on their surface. Dassie et al. further improved this system by optimizing both parts of the chimeric species in order to allow systemic administration, which should simplify clinical applications [226]. The fabrication of the second generation of optimized PSMA-Plk1 chimeras involved a reduction of the length of the aptamers (from 71 down to 39 nucleotides) to facilitate chemical synthesis while introduction of 2′ fluoropyrimidines residues in the longer RNA strand increased serum stability. In addition, the gene silencing activity was enhanced by fine-tuning the siRNA sequence for Dicer recognition, RISC complex formation, and mimicking endogenous miRNA precursors. This optimization included the inclusion of a UU 3′-overhang, engineering of a wobble base pair, swapping of the passenger and guide strands, and introduction of a short stem loop chimera. The second-generation chimeras were found to be active at concentration 50-fold lower than the first generation chimeras and produced a target-specific apoptotic activity [226].

A similar approach was followed in a recent study by Liu et al. where an RNA based aptamer siRNA chimera was engineered to target PSMAs of prostate cancer (PCa) [227]. The bivalent aptamer-dual siRNA chimeric system that was used consisted of an anti-PSMA aptamer [228] connected to an siRNA targeted against the survivin oncogene while the second moiety was composed of the same aptamer but linked to an siRNA for the EGFR gene. Upon binding to prostate cancer cells and internalization, the bivalent chimera was divided into the aptamer and the siRNA parts by digestion of the stem-loops by the Dicer activating RNAi machinery. The siRNAs then inhibited both EGFR and survivin simultaneously by selective mRNA cleavage and ultimately induced apoptosis. Both in vitro and in vivo studies revealed that the combination therapy where two oncogenic pathways are targeted simultaneously is a highly efficient strategy for the eradication of tumor growth and angiogenesis. In another similar approach followed by Jeong et al., DOX was intercalated into a multivalent aptamer-siRNA chimera in order to target multidrug resistant mucin1-overexpressing breast cancer cells (MCF7) [229]. A multimeric antisense siRNA construct was first built by covalent attachment of about 18 single 3′- and 5′-end dithiolated BCL2-specific siRNA sequences through a dithio-bis-maleimidoethane linker and by application of the thiol-ene click reaction. The resulting multivalent template was designed to bind to a chimera comprising a complementary siRNA sequence (the sense strand) and an anti-MUC1 aptamer for the selective delivery of the siRNA to the intended target. DOX was loaded on the construct in the double-stranded regions and the subsequent in vitro tests showed that the DOX-aptamer-siRNA efficiently reduced the viability of the cancer cells after one day by activating the apoptotic caspase-3/7 and releasing DOX as a therapeutic agent. Compared to monovalent DOX-aptamer-siRNA or free DOX, administration of the multivalent complex was the only one that led to a low recovery rate of the cancer cells. The construct thus fulfilled its intended purposes: selective delivery of DOX and efficient antisense activity.

Wilner et al. targeted the transferrin receptor CD71 (TfR) which is overexpressed in malignant cells with a liposome labeled with aptamers and loaded with an anti-enhanced green fluorescent protein siRNA [230]. To achieve specific delivery, they developed a nuclease-resistant aptamer that was obtained through application of a modified SELEX protocol. Indeed, in the first step of the hybrid in vitro selection protocol, a traditional SELEX was carried out using 2′-fluoro-pyrmidine NTPs (in lieu of the natural UTP and CTP) and recombinant hTfR immobilized on a solid support. After the 5th round of selection, a second, “internalization selection”, step was included which consisted of incubation of the enriched RNA libraries stemming from the different generations with HeLa cells and extraction and amplification of the RNA molecules internalized by the cells. This ingenious selection protocol allowed for the isolation of a highly potent RNA aptamer (*K*_d_ = 17 nM) that could efficiently penetrate Jurkat cells and retain its high binding affinity. The selected anti-TfR aptamer could be engineered into a truncated version (*K*_d_ = 102 nM) and was then used to functionalize stable nucleic acid lipid particles (SNALP) containing the siRNA by connection with a thiol maleimide linker. Upon internalization of the functionalized SNALPs, an efficient gene knockdown activity was observed in HeLa cells in vitro, thus providing evidence for the successful activation of the RNAi pathway of the siRNA.

Paralleling these efforts, an elegant approach for aptamer-mediated siRNA delivery was developed by Chu et al. in 2006 in which streptavidin served as a tetravalent core to connect two identical biotinylated anti-PSMA aptamers and two identical biotinylated siRNAs directed against lamin A/C (Figure 6C). In vitro studies with LNCaP cells overexpressing PSMA showed that the chimeric construct was internalized within 30 min and that the gene expression was significantly reduced only when both the siRNA and the anti PSMA aptamers were present on the construct [209]. Control experiments with PSMA-negative PC3 cells demonstrated no cytotoxic effects and no reduction of gene expression, highlighting the potential of such a set up as a gene therapeutic agent despite a potential immunogenicity of the streptavidin adducts which could limit the delivery of such constructs [231]. The same anti-PSMA aptamer was recently involved in the construction of a pRNA-3WJ core based system for the specific delivery of a miRNA LNA to LNCaP prostate cancer cells and to knock down the oncogenes miR17 and miR21 [232]. In addition to the miRNA and the aptamer, a Cy5 dye was introduced in the framework to follow the in vitro internalization into cells. The ultra-stable and serum resistant constructs bound to PSMA in an excellent manner at RNA concentrations as low as 100 nM and were shown to deliver the miRNA specifically to the LNCap cells but not to PC-3 cells. Systemic in vivo administration to xenograft tumors in nude mice showed that the aptamer-pRNA-siRNA construct specifically bound to tumor cells with little or no accumulation in healthy cells. A reduced tumor growth could be found even days post administration without any indication of toxicity as a result of the specific delivery.

Lastly, aptamers have also been linked to catalytic DNA (DNAzymes [4]) and RNA (ribozymes [233]) molecules. For instance, a two-step selection protocol was developed to generate RNA molecules capable of both recognizing and binding to the inteRNAl ribosome entry site (IRES) of hepatitis C virus (HCV) and cleavage of the genomic viral RNA at a specific location [234]. This selection experiment led to the identification of seven distinct groups of aptamer-ribozyme chimeras that selectively bound to the intended target (*K*_d_ values of ~5–200 nM) and cleaved the viral RNA with appreciable rate constants (*k*_obs_ ≅ 0.01–0.04 min^−^^1^) [234,235]. Recently, a partial randomization of the sequence of an isolated aptamer-ribozyme followed by reselection allowed improving the inhibitory properties of such RNA molecules [236].

In the context of catalytic DNA molecules, a hemin/G-quadruplex (hGQ) horseradish peroxidase-mimicking DNAzyme [237] was linked to various aptamers specific for certain small molecules—yielding constructs coined nucleoapzymes—to expand the catalytic repertoire of these biocatalysts [238]. Particularly, when the DNAzyme was conjugated with DA- and arginine-binding aptamers, increased yields of oxidation (with multiple turnover kinetics) could be observed with the respective substrates (i.e., DA and N-hydroxy-L-arginine) compared to the individual functional nucleic acids.

## 6. Recent Chemical Modifications of Aptamers

Aptamers are often referred to as nucleic acids antibodies, however the chemical arsenal of DNA and RNA is rather limited when compared to that of proteins. In addition, unmodified, natural nucleic acids are highly prone to hydrolytic degradation by nucleases. The possibility of using chemical modifications in SELEX might help to alleviate these shortcomings [239]. Over the last quarter century, it was shown by several groups that incorporation of nucleoside triphosphates modified at the level of the α-phosphate, the sugar scaffold (mainly at the 2′ position), or at the level of the nucleobase (5 position of pyridines or 7 position of purines) can enhance the target affinity as well as the serum stability compared to aptamers that are restricted to natural nucleotides. Since the principle of using modified nucleotides in SELEX is a known strategy that has been reviewed extensively [240,241,242,243,244], this section will only highlight recent advances in this field.

### 6.1. Base Modified Aptamers

**Figure 7:**
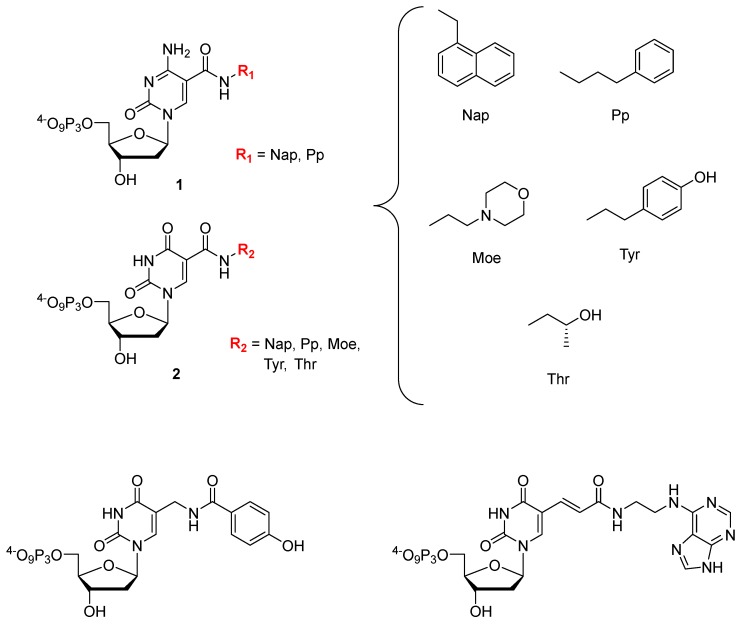
Chemical structures of base-modified nucleoside triphosphates used in selection experiments of aptamers with an expanded chemical repertoire.

Gawande et al. recently investigated the influence on the outcome of a selection process when using a library comprising two amino acid-like 5′-modified pyrimidine bases (dC^X^ 1, dU^X^; Figure 7) or a similar population of oligonucleotides but prepared with only one base modification [245]. A systematic study with all the possible pairwise combinations (i.e., 18 different libraries) of dCTPs equipped with two different side-chains (Nap and Pp) and five different modifications on dUTP (Nap, Tyr, Moe, Thr, and Pp) to find high affinity ligands for proprotein convertase substilisin/kexin type 9 (PCSK9) showed that after six rounds of selection, the libraries made with a single dN*TP were enriched with the modification while libraries prepared with two modifications displayed an increased content of the modified dC^X^ only. Ligands that showed a high affinity for PCSK9 with two modifications were in general more frequent than ligands with only one modification, with the selection experiments exploiting the combination of Tyr-dU and Pp-dC or Nap-dC performing best. A synergistic behavior of the two modifications with respect to affinity could be observed in some cases when compared to aptamers containing only one of the modifications separately. Interestingly, a useful property of double modified aptamers is the high abundance of the modified nucleotides within the sequence. It could be demonstrated that this allows truncating the aptamers with a lower loss of functionality compared to aptamers bearing only one modification. In addition, the serum stability was higher for SOMAmers with two distinct modification compared to natural or ligands with only a single modification. Lastly, another favorable property of using multiple modifications is a higher epitope coverage compared to natural aptamers or ligands containing only a single modification.

With the rationale of using nucleobases equipped with an amino acid-like modification to mimic the hypervariable domains of certain antibodies where tyrosine is overrepresented, Perrin et al. used a phenol modified 5′-deoxyuridine triphosphate (d^y^UTP) [246] to raise aptamers against *E. coli* DH5α cells [247]. Despite the lower incorporation efficiency by the Vent (*exo*^−^) DNA polymerase of d^y^UTP compared to natural dTTP, a relative high abundance of the modified nucleotide (much larger than if introduced randomly) was found in the ligands obtained and sequenced after 12 rounds of a whole cell-SELEX. Unexpectedly, sequencing of the enriched library revealed a vast diversity of sequences that could not be classified into families, thus showing a low abundance of sequence identity. It was hypothesized that this lack of sequence identity was caused by the vast diversity of targets expressed on the cell surface of such a bacteria. Analysis of the binding specificity of the four most abundant aptamers compared with an unmodified aptamer as control for the gram positive cells showed that: (1) the modification is required for high affinity binding; and (2) that cross-reactivity with other cell strains was minimal. The apparent dissociation constant of the most promising aptamer was determined by saturation experiments to be 27.4 nM, which is about 10-fold lower than unmodified RNA aptamers recently reported for DH5α cells [248].

Minagawa et al. used a base-appended base (BAB) in a 75 nucleotide long modified library with a 30 mer randomized region to select aptamers against salivary α-amylase (sAA) [249]. Indeed, replacement of the thymidine nucleotide with a triphosphate displaying an adenine attached to the C5 position of the nucleobase (dU^ad^TP [250], Figure 7) in the selection protocol led to the isolation of seven aptamers against sAA with an abundance of over 5% of the enriched pool after eight rounds of selection. SPR measurements revealed that the most potent aptamer bound to the target with an affinity of 559 pM, which is sufficient for potential applications as a biosensor of the stress biomarker sAA [251]. When the selection experiment was carried out in the absence of dU^ad^TP (only natural dNTPs), no enrichment of the library was observed, further highlighting the usefulness and the potential of modified nucleoside triphosphates in SELEX. An optimization study of the initial 75 mer aptamer showed that the full-length sequence could be truncated down to a 36 mer species without inducing a substantial loss of binding affinity. Additionally, imino-proton NMR spectra were recorded at different temperatures to elucidate the structural properties of the sAA binding aptamer. This NMR analysis suggested that the imino-protons of the adenine part of dU^ad^ were engaged in additional hydrogen-bonding interactions, thus leading to the assumption that the BAB modification induced a well-defined and compact secondary structure. Finally, the potential of the selected aptamer was highlighted with the detection of human sAA in human saliva using capillary electrophoresis, pull down, and lateral flow assays.

Examples of base-modified RNA aptamers are not as common as for DNA, which reflects both the lower tolerance of natural RNA polymerases for base-modified NTPs and the restricted choice of engineered RNA polymerases. Despite these limitations, allyl-amino-UTP (U^aa^TP) was used in an in vitro selection experiment aimed at raising an anti-ATP aptamer [252]. More recently, Kabza and Sczepanski used the same U^aa^TP to isolate an aptamer against the oncogenic precursor microRNA 19a (pre-miR-19a) which was converted to its Spiegelmer via solid-phase synthesis using the L-U^aa^-phosphoramidite [253]. The resulting L-RNA aptamer bound its target with a slightly lower affinity than its D-counterpart (*K*_d_ values of 2.2 and 0.72 nM, respectively) but efficiently inhibited the Dicer-mediated processing of the miR target (IC_50_ ≅ 4 nM) in vitro.

Lastly, the Hili laboratory is currently exploring an interesting approach—coined LOOPER (Ligase-catalyzed OligOnucleotide PolymERization)—for the increase of chemical diversity without the involvement of dN*TPs and relying on the ligation of base-modified pentanucleotides [254,255]. This strategy was recently expanded to the Darwinian evolution of aptamers against human α-thrombin [256]. Indeed, a library containing 16 different pentanucleotidic codons (and as many functional groups ranging from hydrophobic to Brønsted acids and bases) was subjected to six rounds of SELEX. The most highly represented sequence displayed a remarkable affinity (*K*_d_ = 1.6 nM) and selectivity for its target (no cross-reactivity with BSA) and strictly required the presence of the modifications for binding. In addition to ablating the need for dN*TPs (and to an extent engineered polymerases), this strategy allows for the introduction of a broad variety of functional groups and will certainly be used for the evolution of other aptamers and potentially DNAzymes.

### 6.2. Aptamers with an Extended Genetic Alphabet

**Figure 8:**
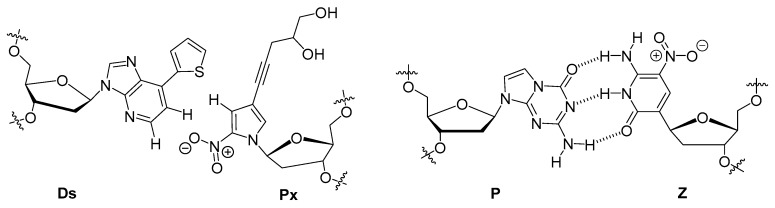
Chemical structures of the unnatural base pairs Ds Px and dP dZ used in the expansion of the genetic code.

Expanding the genetic code from a two- to a three- or even a four-base-pair system is a long standing goal in synthetic biology since this would enable the creation of functional nucleic acids with improved properties and ultimately to semi-synthetic organisms with proteins that potentially display novel structures and/or functions [257]. In this context, Hirao and colleagues have developed the ex-SELEX (genetic alphabet expansion for systematic evolution of ligands by exponential enrichment) method where an additional artificial Ds-Px base pair (Figure 8) complements the two natural Watson–Crick base pairs to isolate aptamers against the vascular endothelial growth factor (VEGF_165_) [258,259]. In the ex-SELEX strategy, the hydrophobic base Ds is introduced into DNA by solid-phase synthesis at predetermined locations and specifically binds to its unnatural partner Px in PCR for the amplification of the generations during SELEX. The localization of the Ds modifications in each sublibrary—a key step in the ex-SELEX protocol—is possible through the combination of a unique barcode system and replacement PCR. In a new approach aimed at exploring a larger chemical space, a fully randomized library containing the unnatural base was used to isolate aptamers targeting the Willebrand factor A1 domain (vWF) [260]. Although the selection with the randomized additional base in the sequences led to higher affinity ligands compared to the selection with natural base pairs or the ex-SELEX selection procedure with limited sublibraries, it also increased the complexity of the system and led to scaling problems. The new complexity and the possibility of misincorporation of the Ds base during PCR experiments required the introduction of a novel sequencing method to elucidate the exact location of the Ds modification. The authors took advantage of the fact that only dATP misincorporated opposite Px by the Taq polymerase but none of the dye-carrying 2′,3′-dideoxynucleoside-5′-triphosphates were incorporated at these positions, which led to the formation of a gap in the sequencing peak pattern at the position of the unnatural base. By comparing the sequencing pattern with that of a clone after a replacement PCR, the exact positions of the modifications could be inferred. Lastly, both the serum stability and the thermostability of the anti-vWF aptamers could be enhanced by introducing additional mini-hairpin motifs containing GNA loops (N = A, G, C or T) [261] without a loss of affinity in both the natural (*K*_d_ = 182 pM) and the modified aptamer (*K*_d_ = 61 pM), which thus represents a convenient strategy for the improvement and construction of functional aptamers [259,262,263].

In the context of an expansion of the genetic alphabet, Benner and co-workers developed a third base pair dZ-dP (Figure 5) that can be used in SELEX and the related strategy was coined “laboratory in vitro evolution based on an artificial expanded genetic information system” (LIVE-AEGIS) [264,265]. Initial selection experiments using a 20 nucleotide long randomized library with a nucleotide composition of T/G/A/C/Z/P ≅ 1.5/1.2/1.0/1.0/1.0/0.5 by solid-phase synthesis and amplified in the selection rounds Hot Start Taq polymerase in the presence of natural and modified dNTPs led to the isolation of a high affinity aptamer (*K*_d_ = 30 nM) against MDA-MB 231 breast cancer cells [266]. In order to perform deep sequencing of the enriched populations at the end of the LIVE-AEGIS protocol, a conversion technique was used where the dZ nucleotides were converted into dC and dT and dP into dA and dG, allowing to assign the positions of the two modifications. Analysis of the sequence composition showed that only one dZ was present in the most potent aptamer which additionally lost its binding affinity when the modification was replaced with a natural nucleotide. The depletion of dZ and dP nucleotides in the isolated aptamer (an average of three dZ and 1.5 dP would be expected for a 20 nucleotide long sequence) was ascribed to a slight disadvantage of the modified triphosphates during PCR compared to the natural nucleotides. A similar observation was made in another study by Zhang et al. using the AEGIS system for cells engineered to place glypican 3 (hGPC3) on their surface where a ligand with only one dZ was found that bound with an affinity of 6 nM [267]. Analogues without the dZ showed a significant loss in activity again demonstrating the importance of the modification. The most recent AEGIS-LIVE study by Biondi et al. targeting the protective antigen (PA63), a cleaved version of the precursor protein PA83 from *Bacillus anthracis*, brought forth an aptamer that binds to PA63 and thus blocks the toxin channel by displacing the lethal factor and finally inhibiting translocation of toxins into infected cell [268]. Analysis of the sequences stemming from the AEGIS-LIVE selections revealed that no survivor contained a dZ modification but different dP-containing sequences were isolated [268]. The most abundant sequence of the enriched pool comprised two dP nucleotides and this aptameric species displayed a very high affinity for PA63 (*K*_d_ ~ 35 nM). This underrepresentation of modifications in the surviving sequences was ascribed to the conformational constraint imposed by the modified nucleotides. An intensive analysis of the secondary structure of the aptamer revealed interesting features resembling the formation of a higher folded structure [269] upon addition of cations that was not described in the literature before and which was also found to increase the stability against nuclease degradation.

Other prominent base-modified nucleoside and nucleotide analogs that have not (yet) been used to generate aptamers with enhanced properties include the DNAM-5dSICS unnatural base pair (UBP) developed by the Romesberg laboratory and used in the development of a semi-synthetic organism [270,271], artificial metallo-base pairs [272,273], and nucleotides modified with other functional groups [125,274,275,276,277,278].

### 6.3. Sugar Modified Aptamers

**Figure 9:**
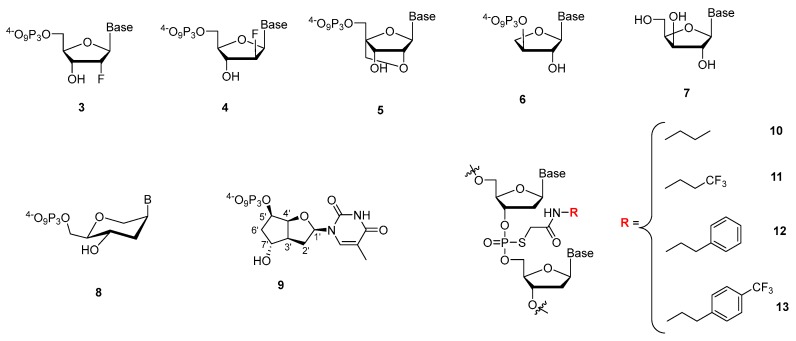
Chemical structures of sugar and phosphate modified nucleotides.

2′-fluoro-nucleotides (**3** in Figure 9) are popular modifications that are often introduced into aptameric scaffolds to increase their nuclease resistance, a strategy that culminated in the development of pegaptanib sodium (Macugen^®^) [25,240]. However, 2′-fluoro-modified nucleoside triphosphates are rather poor substrates for DNA polymerases which restricts their use in selection experiments and often require a time consuming reverse transcription in order to convert the 2′-modified sequences into natural oligonucleotides that can be amplified and then transcribed back into modified sequences [279]. In order to circumvent these shortcomings, the Romesberg laboratory evolved a thermostable polymerase that could PCR-amplify oligonucleotides containing 2′-fluoro- and 2′-OMe-nucleotides [280]. This polymerase, SFM4-3, was then recently used in a selection experiment with 2′-fluorine-modified purine nucleotides to raise aptamers that could bind human neutrophil elastase (NHE) [281]. With the engineered thermostable DNA polymerase SFM4-3, the need for a reverse transcription step was ablated which significantly shortened and simplified the selection process. After six rounds of selection, two aptamers were identified that displayed very high affinity (*K*_d_ = 20–170 nM) for the NHE target, albeit with slightly lower affinity than that of the sequences that did not contain the 2′-fluoro-modifications (*K*_d_ = 11–17 nM). In order to exclude nonspecific electrostatic interactions of the aptamers with the positively charged HNE, an experiment suppressing the charges with high salt concentrations (i.e., 1 M NaCl) was performed, where the natural variant of the aptamers lost its affinity to HNE but the ligand with the 2′-fluoro modification remained bound. Lastly, ^19^F NMR experiments revealed that the secondary structure of the aptamer was influenced by the presence of the 2′-modifications which disfavours duplex formation and therefore allows the formation of a more soluble species that specifically recognized the target. A similar selection experiment was carried out to isolate fully-modified 2′-OMe-aptamers by combining an engineered polymerase and a reverse transcriptase. One particular aptamer, 2mHNE-4, bound its intended target, human neutrophil elastase, with good affinity (K_d_ = 45 nM) [282].

Other sugar modifications such as HNA (hexitol nucleic acid **8**) [283], LNA (locked nucleic acid **5**) [283], FANA (2′-fluoro-arabinose **4**) [284], and TNA (threose nucleic acid **6**) [285] have all been used for the generation of highly potent modified aptamers, while other nucleotides such as 7′,5′-bicyclo-DNA **9** [286], xylonucleic acids **7** [287], 2′-selenomethyl nucleotides [288], and 4′thio-DNA [289,290] have recently been suggested as potential candidates to explore new chemistries in selection experiments (Figure 9) [279,291].

### 6.4. Phosphate Modified Aptamers

The possibilities for the modification at the level of the phosphate unit are more limited than in the case of the sugar and nucleobases moieties and most efforts have focused on the α-phosphate, particularly α-phosphorothioates [242,279,292,293,294]. In this context, Yang et al. have explored the alkylation of phosphorothioated thrombine-binding aptamers (TBA) with aim of improving the antitumor properties of the ligands by reducing the thrombin binding affinity [295]. Alkylation of the phosphorothiate moieties was achieved by a simple substitution reaction with four different brominated substrates (**10** to **13** in Figure 9). Previous X-ray [296] and NMR studies [297] of the TBA showed that the TT or TGT loop of the G-quadruplex structure was responsible for effective binding. Therefore, the modifications were systematically introduced at these positions. The presence of the phenyl moiety (**12** in Figure 9) was shown to reduce the flexibility of the loop regions through interaction with nucleobases which resulted in an inhibition of G-quadruplex formation which in turn is believed to cause a lowering of the binding affinity (*K*_d_ = 0.5 μM) for thrombin compared to the unmodified aptamer (*K*_d_ = 0.19 μM). The higher dissociation constants for the aptamers modified with the phenyl moiety also resulted in a reduction of the anticoagulation properties. The antiproliferation experiments with the natural aptamers as control revealed that the TBAs with the phenyl modification exhibited excellent inhibition of the proliferation of about 80% with the lung carcinoma cell line A549 but no activity against the human breast cancer cell line MCF-7.

Substitution of an oxygen atom on the α-phosphate with a BH_3_ moiety instead of a sulfur atom was used for the selection of a potent anti-ATP aptamer but no further examples have been reported since [298].

## 7. Conclusions and Prospects

In view of their impressive functional properties, nucleic acid aptamers certainly deserve their description as chemical antibodies [25]. The high degree of structural flexibility associated with an impressive target specificity and selectivity has propelled aptamers into the forefront of numerous therapeutic and diagnostic applications. Moreover, aptamers are continuously employed in proof-of-concept studies to further expand the boundaries of the realm of aptamer-based technologies. Particularly, aptamers have recently started to infiltrate the fields of medical imaging and there is no doubt that aptamers will mature into valuable and smart imaging agents. In addition, aptamers commence to be used as tools in neuroscience for the detection of the variation of small concentrations of various neurotransmitters and abnormal protein folds. Hopes for the in vivo use of aptamers in neurosciences are spurred by a recent selection experiment of an aptamer for its capacity at penetrating the blood–brain barrier (BBB) [299] as well as a novel bioconjugation method to polymeric nanoparticles which has allowed the in vivo BBB passage [300]. Furthermore, due to their chemical malleability, aptamers can easily be conjugated to small molecules, other nucleic acid oligonucleotides, or larger constructs such as antibodies, liposomes, and DNA origamis that undoubtedly will improve their cellular uptake and help in the development of aptamer-mediated drug delivery systems as well as biosensing platforms. However, aptamers still suffer from temperature- and nuclease-mediated degradation and the restricted chemical arsenal available to natural nucleic acids precludes binding to difficult targets such as single enantiomers of small organic molecules or glycosylated proteins [240,301]. The use of modified nucleoside triphosphates, along with engineered polymerases [291,302] and new selection strategies [256,285,303,304,305], will certainly help in alleviating these predicaments.

All the emerging applications described in this Review along with technological and synthetic progress will certainly improve the limited commercial success of aptamers in the near future.

## Abbreviations

CTC	Circulating Cancer Cells
GO	Graphene oxide
AFM	Atomic Force Microscopy
SELEX	Systematic Evolution of Ligands by Exponential Enrichment
PET	Positron Emission Tomography
MRI	Magnetic Resonance Imaging
DOTA	1,4,7,10-tetraazacyclododecane-1,4,7,10-tetraacetic acid
*K*_D_	Dissociation constant
XNA	Xeno nucleic acid
SPIONs	Superparamagnetic iron oxide nanoparticles
NOTA	1,4,7-triazacyclononane-triacetic acid
DNAzyme	DNA enzyme or catalytic DNA
SPECT	Single Photon Emission Computed Tomography
HER2	Human Epidermal Growth Factor Receptor 2
DOX	Doxorubicin
QD	Quantum Dot
PEG	Polyethyleneglycol
siRNA	Small interfering RNA
miRNA	Micro RNA
sgRNA	Single guide RNA
pRNA	Packaging RNA
3WJ	Three-way junction
GABA	γ-Aminobutyric acid
BSA	Bovine Serum Albumin
Aβ	Amyloid β
